# Supplementary material for: Members of an array of zinc finger proteins specify distinct Hox chromatin boundaries
Source: Mol Cell. Author manuscript; Available in PMC 2024 Dec 3. (PMC11613955; doi:10.1016/j.molcel.2024.08.007)
Supplement: 1 [file NIHMS2020155-supplement-1.pdf]

Supplementary Materials for

Members of an array of zinc finger proteins specify distinct *Hox* chromatin boundaries

Havva Ortabozkoyun[1,2,3,4,†,*], Pin-Yao Huang[1,4,†], Edgar Gonzalez-Buendia[1,2,3,4], Hyein Cho[5,6,7], Sang Y. Kim[5], Aristotelis Tsirigos[5,6,8], Esteban O. Mazzoni[9], Danny Reinberg[1,2,3,4,10,*]

[*] Correspondence to: dxr1274@miami.edu, hxo128@miami.edu

This PDF file includes:

Figures S1 to S11

SUPPLEMENTARY FIGURES

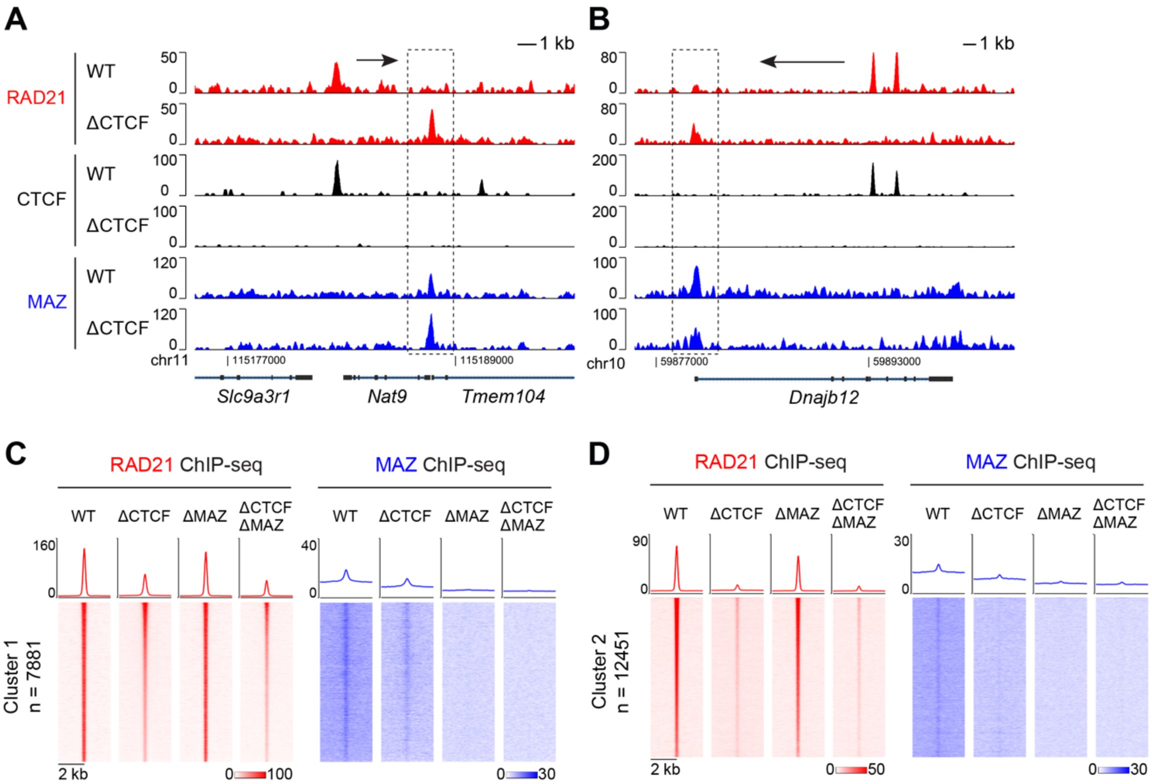

E

Motif (MEME)	E-value	Sites	Top 10 Matches (Tomtom) Mouse		Human	
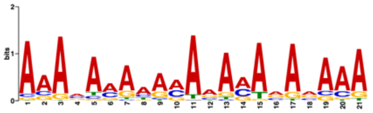	4.3e-128	1000	STAT1	ONEC2	CPEB1	FOXG1
			FUBP1	IRF5	PRDM6	STAT1
			FOXJ3	STAT2	FUBP1	FOXJ3
			IRF3	TCF7	NFAC1	IRF1
			FOXJ3	IRF1	FOXL1	ANDR
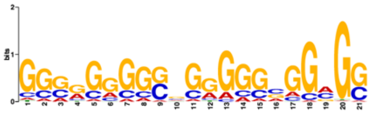	1.9e-098	1000	SP2	MAZ	SP2	ZN467
			SP5	ZBT17	PATZ1	MAZ
			SP3	KLF15	SP3	ZN263
			WT1	KLF3	WT1	VEZF1
			SP1	ZN281	ZN341	TBX15
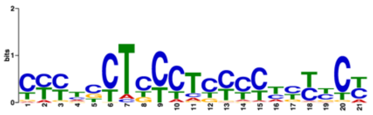	4.3e-024	879	SP5	KLF15	VEZF1	PATZ1
			MAZ	SP4	ZN467	FLI1
			WT1	ELF5	ZN341	ZN263
			FLI1	SP3	MAZ	KLF15
			SP5	ETS2	WT1	RXRA
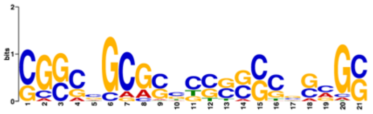	5.8e-004	440	SP2	KLF3	SP2	SP1
			ZFX	SP4	SP1	KLF9
			SP3	SP1	SP3	THAP1
			SP1	ZBT17	AP2D	SP4
			AP2D	MXI1	KLF3	KLF12

Figure S1. Loss of MAZ in CTCF-degraded mESCs results in the reduction of re-localized RAD21 signal at regions co-occupied by MAZ and RAD21, related to Figure 1

(A-B) Normalized ChIP-seq densities for RAD21, CTCF, and MAZ at (A) *Nat9* and (B) *Dnajb12* loci where re-localized RAD21 overlaps with MAZ binding. ChIP-seq data is from one representative of two biological replicates for CTCF and MAZ, and one biological replicate for RAD21. Additional RAD21 ChIP-seq datasets were reported earlier[1,2]. RAD21-relocalized regions are indicated within dashed-lines. Arrows indicate proximal RAD21 peak to indicate the possible re-localization.

(C-D) Heat maps of RAD21 and MAZ ChIP-seq read density in (C) Cluster 1 (n=7,881) and (D) Cluster 2 (n=12,451) within a 4 kb window in WT, ΔCTCF, ΔMAZ, and ΔCTCF/ΔMAZ conditions in ESCs. Average density profiles for ChIP-seq under each condition is indicated above the heat map. ChIP-seq data is from one representative biological replicate for CTCF degron ESCs and one representative of two biological replicates for MAZ KO clones in CTCF degron background.

(E) Motif identification for re-localized RAD21 peaks in the absence of CTCF and MAZ by *de novo* MEME motif analysis, along with the corresponding top matches by Tomtom motif comparison tool. Motif search in MEME has been performed *de novo* until 1000 sites were reached and corresponding e-values are depicted in the table.

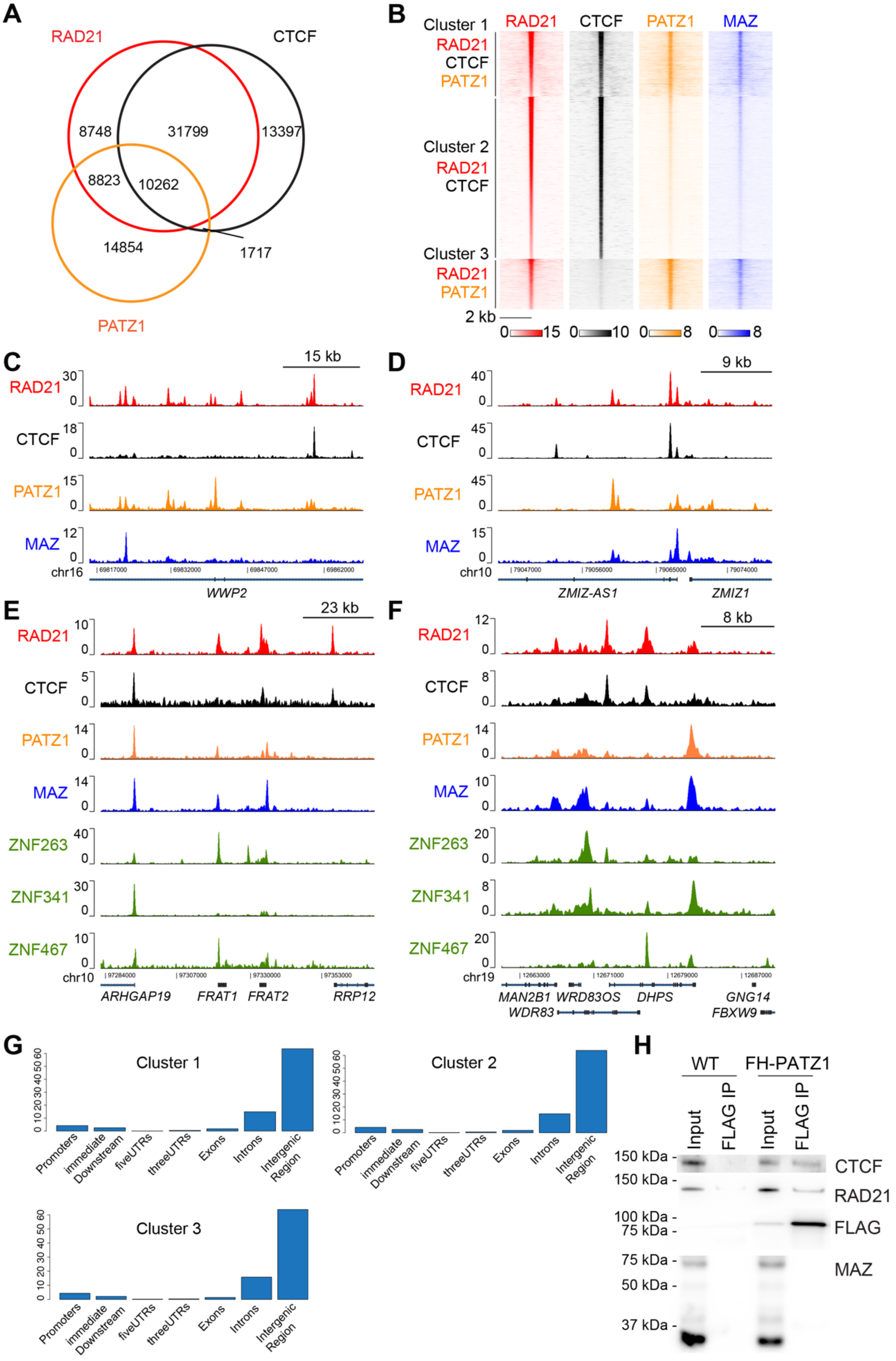

A
RAD21
CTCF
PATZ1
8748
31799
13397
8823
10262
14854
1717
B
Cluster 1
RAD21
CTCF
PATZ1
Cluster 2
RAD21
CTCF
Cluster 3
RAD21
PATZ1
MAZ
2 kb
C
15 kb
RAD21
CTCF
PATZ1
MAZ
chr16
WWP2
D
9 kb
chr10
ZMIZ-AS1
ZMIZ1
E
23 kb
ZNF263
ZNF341
ZNF467
ARHGAP19
FRAT1
FRAT2
RRP12
F
8 kb
chr19
MAN2B1
WRD83OS
DHPS
GNG14
WDR83
FBXW9
G
Cluster 1
Cluster 2
Cluster 3
Promoters
immediate Downstream
fiveUTRs
threeUTRs
Exons
Introns
Intergenic Region
H
WT
FH-PATZ1
Input
FLAG IP
150 kDa
100 kDa
75 kDa
50 kDa
37 kDa
FLAG
MAZ

Figure S2. PATZ1 co-localizes with RAD21 on chromatin in HepG2 and HEK293 cells, related to Figure 2

(A) Venn diagram showing RAD21, CTCF, and PATZ1 binding in HepG2 cells.

(B) Heat maps of RAD21, CTCF, PATZ1, and MAZ ChIP-seq read density in HepG2 cells clustered as Cluster 1, Cluster 2, and Cluster 3 based on the indicated overlaps with RAD21 signal within a 4 kb window.

(C-D) Normalized ChIP-seq densities for RAD21, CTCF, PATZ1, and MAZ, wherein peaks co-localizing with RAD21 and/or CTCF were visualized in HepG2 cells. ChIP-seq data in HepG2 cells is from two combined biological replicates.

(E-F) Normalized ChIP-seq densities for RAD21, CTCF, PATZ1, MAZ, and other zinc finger proteins, ZNF263, ZNF341, and ZNF467, wherein RAD21 and/or CTCF co-localizing peaks were observed in HEK293 cells. ChIP-seq data in HEK293 cells is from one replicate for RAD21 and one representative of two biological replicates for others. The source of genomics data used is listed in Table S1.

(G) Distribution of RAD21, CTCF, and PATZ1 co-localizing binding sites across genomic features based on Figure 2B. Cluster 1, 2, and 3 refers to the clusters shown in Figure 2B.

(H) Western blot analysis of CTCF, RAD21, FLAG, and MAZ upon FLAG-PATZ1 immunoprecipitation from nuclear extract of 293FT cells (n=2, see Figure 2C).

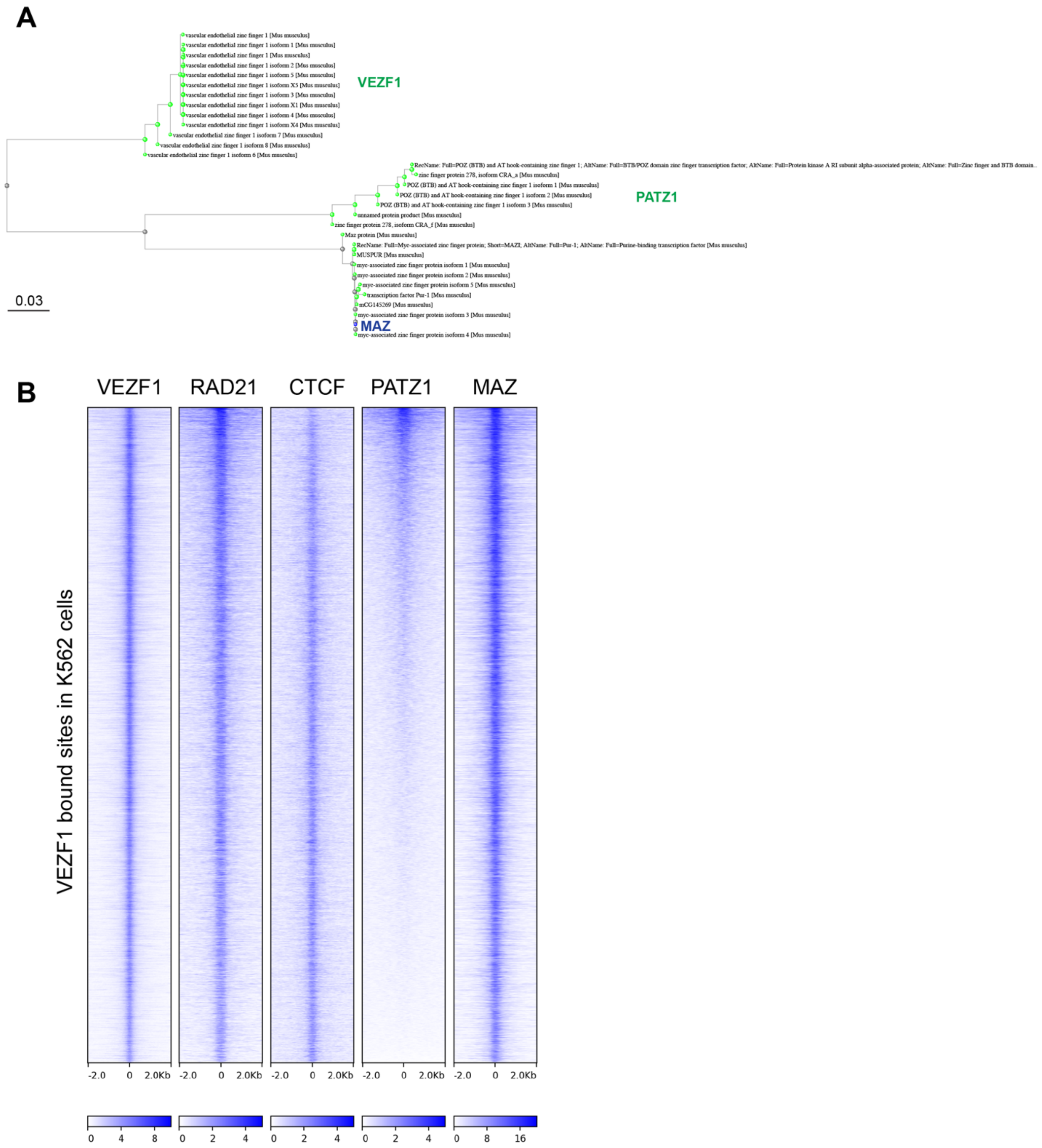

Figure S3. PATZ1 and VEZF1 show protein sequence similarity to MAZ, and co-localize with MAZ, related to Figure 1 and 2

(A) Protein blast (blastp) analysis of MAZ protein. Top 30 matches were drawn as the tree.

(B) Heat maps of VEZF1, RAD21, CTCF, PATZ1 and MAZ ChIP-seq read density in VEZF1 binding sites within a 4 kb window in K562 cells. ChIP-seq data in K562 cells is from one representative of two biological replicates. The source of genomics data used is listed in Table S1. VEZF1 ChIP-seq was re-analyzed.

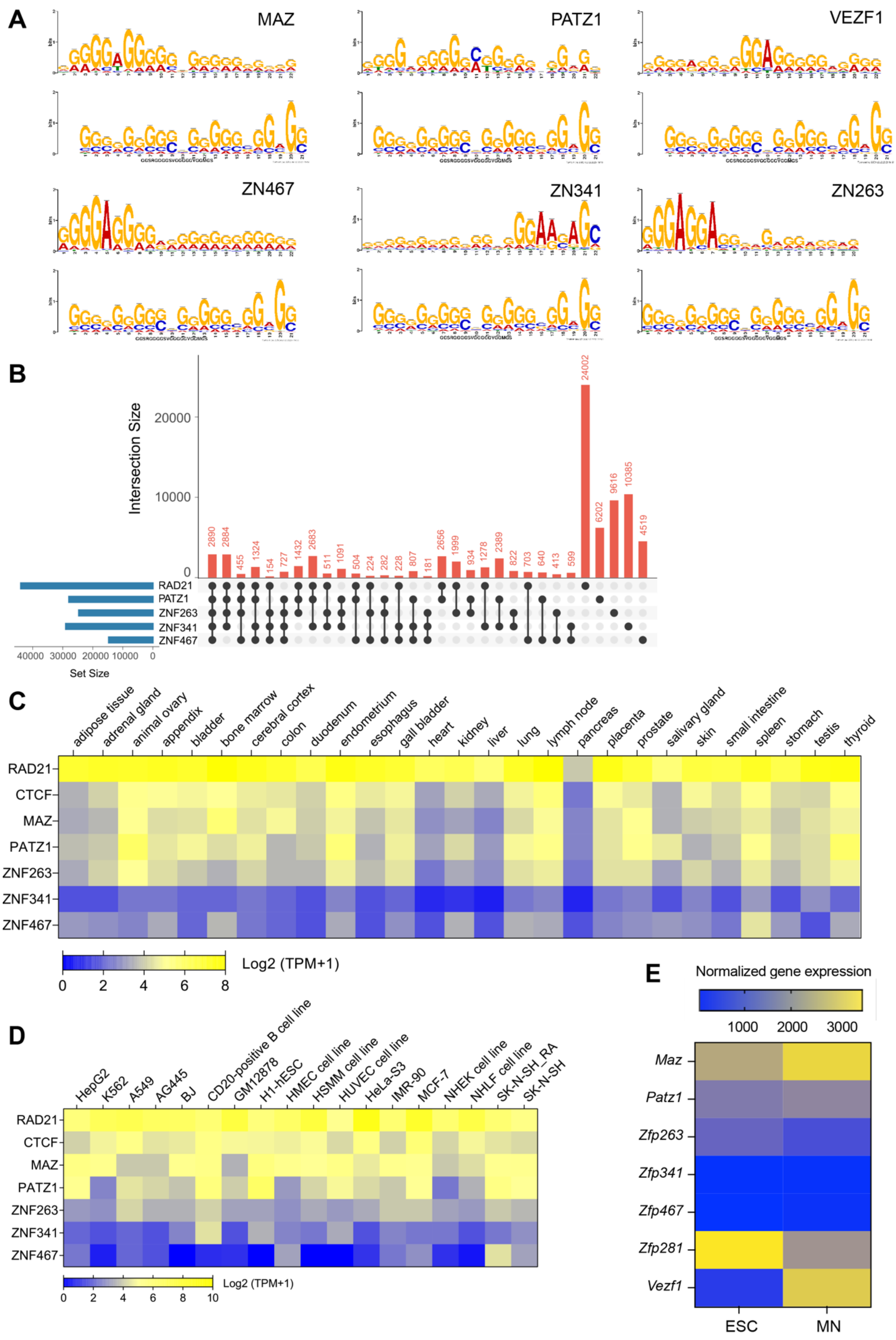
A
MAZ
PATZ1
VEZF1
ZN467
ZN341
ZN263
B
Intersection Size
20000
10000
0
2890
2884
455
1324
154
727
1432
2683
511
1091
504
224
282
228
807
181
2656
1999
934
1278
2389
822
703
640
413
599
24002
6202
9616
10385
4519
RAD21
PATZ1
ZNF263
ZNF341
ZNF467
40000 30000 20000 10000 0
Set Size
C
adipose tissue
adrenal gland
animal ovary
appendix
bladder
bone marrow
cerebral cortex
colon
duodenum
endometrium
esophagus
gall bladder
heart
kidney
liver
lung
lymph node
pancreas
placenta
prostate
salivary gland
skin
small intestine
spleen
stomach
testis
thyroid
RAD21
CTCF
MAZ
PATZ1
ZNF263
ZNF341
ZNF467
0 2 4 6 8
Log2 (TPM+1)
D
HepG2
K562
A549
AG445
BJ
CD20-positive B cell line
GM12878
H1-hESC
HMEC cell line
HSMM cell line
HUVEC cell line
HeLa-S3
IMR-90
MCF-7
NHEK cell line
NHLF cell line
SK-N-SH_RA
SK-N-SH
RAD21
CTCF
MAZ
PATZ1
ZNF263
ZNF341
ZNF467
0 2 4 6 8 10
Log2 (TPM+1)
E
Normalized gene expression
1000 2000 3000
Maz
Patz1
Zfp263
Zfp341
Zfp467
Zfp281
Vezf1
ESC
MN

Figure S4. PATZ1 and other ZNFs are expressed at varying levels across different tissues and cell types and co-localize with RAD21 on chromatin, related to Figure 1 and 2

(A) Representative alignment of top motif matches to the candidates: MAZ, PATZ1, VEZF1, ZN467, ZN341, and ZN263. *De-novo* motif analysis was performed as depicted in Figure S1E for re-localized RAD21 peaks in the absence of CTCF and MAZ. Motif alignments were generated through Tomtom motif comparison tool.

(B) UpSet plot indicating the overlap of RAD21, PATZ1, ZNF263, ZNF341, and ZNF467 binding in HEK293 cells (see Figure 2F).

(C-D) Heat map of RNA-seq expression [$\log_2$ (TPM+1)] of RAD21 and ZNFs across different human tissues (C) and ENCODE cell lines (D).

(E) Heat map of RNA-seq expression (Normalized counts) of MAZ, PATZ1, ZNFs and VEZF1 during ESC to MN differentiation from two biological replicates.

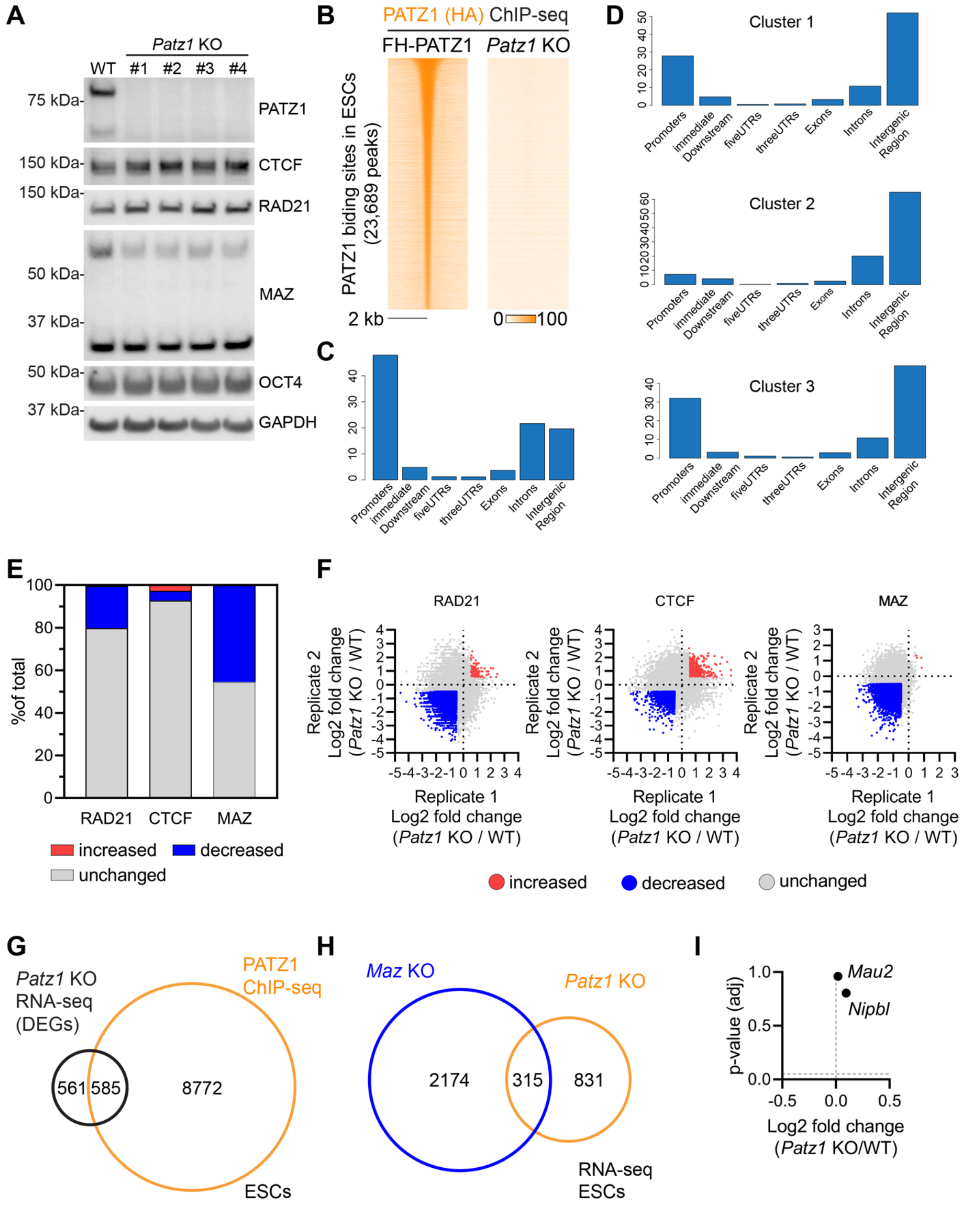
A
Patz1 KO
WT #1 #2 #3 #4
75 kDa-
PATZ1
150 kDa-
CTCF
150 kDa-
RAD21
50 kDa-
37 kDa-
MAZ
50 kDa-
OCT4
37 kDa-
GAPDH
B
PATZ1 (HA) ChIP-seq
FH-PATZ1
Patz1 KO
PATZ1 biding sites in ESCs (23,689 peaks)
2 kb
0 100
C
Promoters
immediate Downstream
fiveUTRs
threeUTRs
Exons
Introns
Intergenic Region
D
Cluster 1
Cluster 2
Cluster 3
E
%of total
RAD21 CTCF MAZ
increased
decreased
unchanged
F
RAD21
CTCF
MAZ
Replicate 2 Log2 fold change (Patz1 KO / WT)
Replicate 1 Log2 fold change (Patz1 KO / WT)
increased
decreased
unchanged
G
Patz1 KO RNA-seq (DEGs)
PATZ1 ChIP-seq
561
585
8772
ESCs
H
Maz KO
Patz1 KO
2174
315
831
RNA-seq ESCs
I
Mau2
Nipbl
p-value (adj)
Log2 fold change (Patz1 KO/WT)

Figure S5. The effects of *Patz1* depletion in mESCs, related to Figures 3 and 4

(A) Western blot analysis of PATZ1, CTCF, RAD21, MAZ, OCT4, and GAPDH in WT and *Patz1* KO clones.

(B) Heat maps of PATZ1 (HA) ChIP-seq read densities in FH-PATZ1 and *Patz1* KO mESCs at PATZ1 peaks, showing the specificity of PATZ1 ChIP-seq signals.

(C) Distribution of PATZ1 binding sites in ESCs across genomic features.

(D) Distribution of RAD21, CTCF, and PATZ1 co-localizing binding sites across genomic features based on Figure 3D. Cluster 1, 2, and 3 refers to the clusters shown in Figure 3D.

(E) Changes in ChIP-seq densities of RAD21, CTCF, and MAZ in *Patz1* KO with the cutoff of ± 0.5 $\log_2$(fold change) observed in two independent biological replicates.

(F) Ratio of RAD21, CTCF, and MAZ ChIP-seq densities between *Patz1* KO and WT from 2 independent biological replicates.

(G) Overlap of PATZ1 ChIP-seq signal with the differentially expressed genes (DEGs) in *Patz1* KO ESCs compared to WT ESCs.

(H) Venn diagram indicating the overlap of differentially expressed genes upon *Patz1* KO and *Maz* KO in mESCs from three biological replicates.

(I) The fold change in expression of the cohesin loader genes, *Nipbl* and *Mau2*, in mESCs was assessed by RNA-seq from three biological replicates.

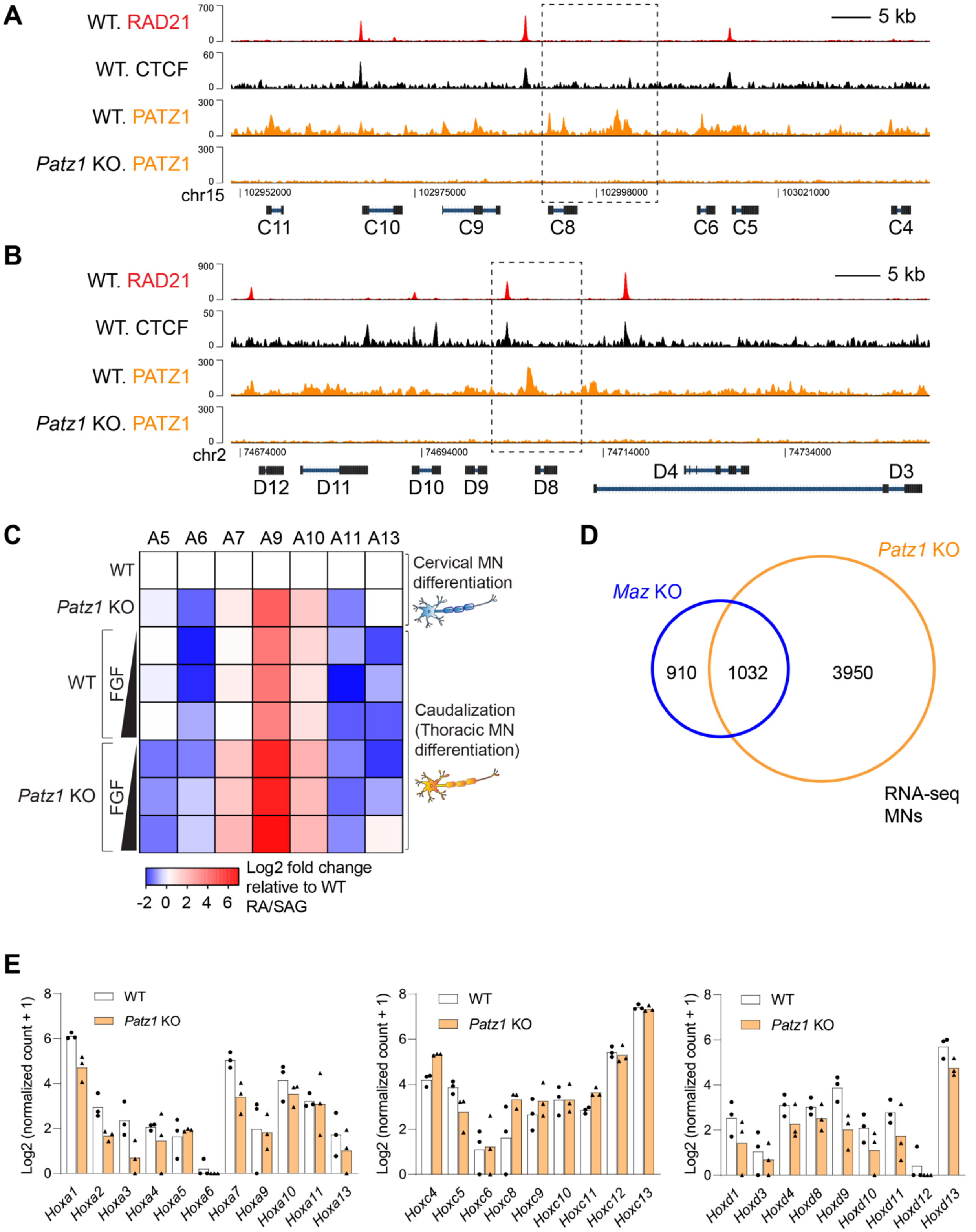

A
WT. RAD21
WT. CTCF
WT. PATZ1
Patz1 KO. PATZ1
5 kb
chr15
102952000
102975000
102998000
103021000
C11
C10
C9
C8
C6
C5
C4
B
WT. RAD21
WT. CTCF
WT. PATZ1
Patz1 KO. PATZ1
5 kb
chr2
74674000
74694000
74714000
74734000
D12
D11
D10
D9
D8
D4
D3
C
A5 A6 A7 A9 A10 A11 A13
WT
Patz1 KO
FGF
Cervical MN differentiation
Caudalization (Thoracic MN differentiation)
Log2 fold change relative to WT RA/SAG
-2 0 2 4 6
D
Maz KO
Patz1 KO
910
1032
3950
RNA-seq MNs
E
Log2 (normalized count + 1)
WT
Patz1 KO
Hoxa1 Hoxa2 Hoxa3 Hoxa4 Hoxa5 Hoxa6 Hoxa7 Hoxa9 Hoxa10 Hoxa11 Hoxa13
Hoxc4 Hoxc5 Hoxc6 Hoxc8 Hoxc9 Hoxc10 Hoxc11 Hoxc12 Hoxc13
Hoxd1 Hoxd3 Hoxd4 Hoxd8 Hoxd9 Hoxd10 Hoxd11 Hoxd12 Hoxd13

Figure S6. Loss of PATZ1 results in de-repression of *Hoxa9*, *Hoxc9* and *Hoxd9* in cervical MNs, indicating a rostro-caudal patterning defect in MNs, related to Figure 5

(A-B) Normalized ChIP-seq densities for RAD21, CTCF, and PATZ1 in WT and *Patz1* KO mESCs at the indicated region in the *HoxC* (A) and *HoxD* (B) cluster.

(C) Heat map of relative gene expression in WT versus *Patz1* KO MNs at the *HoxA* cluster in cervical MNs and thoracic MNs from one biological replicate. WNT/FGF signaling gradient was utilized for MN caudalization, as described previously (see Methods for details).

(D) Venn diagram indicating the overlap of differentially expressed genes upon *Patz1* KO and *Maz* KO in motor neurons from two and three biological replicates, respectively.

(E) RNA-seq normalized counts for *Hox* gene expression across *HoxA, C,* and *D* clusters in WT versus *Patz1* KO ESCs from three biological replicates.

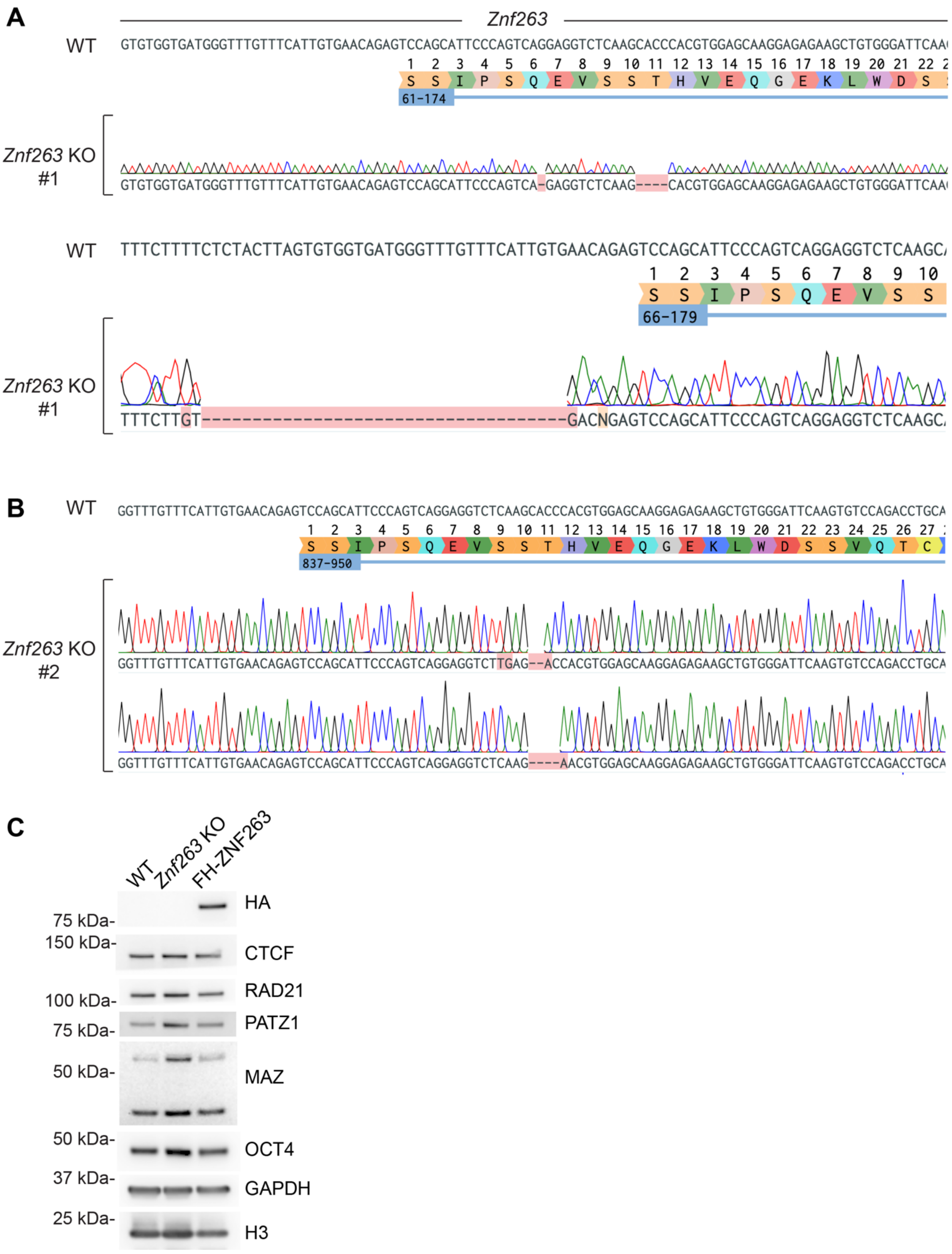
A
Znf263
WT
Znf263 KO #1
61-174
66-179
B
Znf263 KO #2
837-950
C
WT
Znf263 KO
FH-ZNF263
HA
CTCF
RAD21
PATZ1
MAZ
OCT4
GAPDH
H3
75 kDa-
150 kDa-
100 kDa-
50 kDa-
37 kDa-
25 kDa-

Figure S7. *Znf263* KO mESC generation via CRISPR and FH-ZNF263 expression in mESCs, related to Figure 5

(A-B) CRISPR based deletions in two mESC clones upon targeting of the *Znf263* locus (see Figure 5). Clone #1 (A) and clone #2 (B) harbor the indicated deletions resulting in frame-shift mutations. (C) Western blot analysis of FH-ZNF263 (HA), CTCF, RAD21, PATZ1, MAZ, OCT4, GAPDH, and Histone H3 in WT, *Znf263* KO, and FH-ZNF263 mESCs.

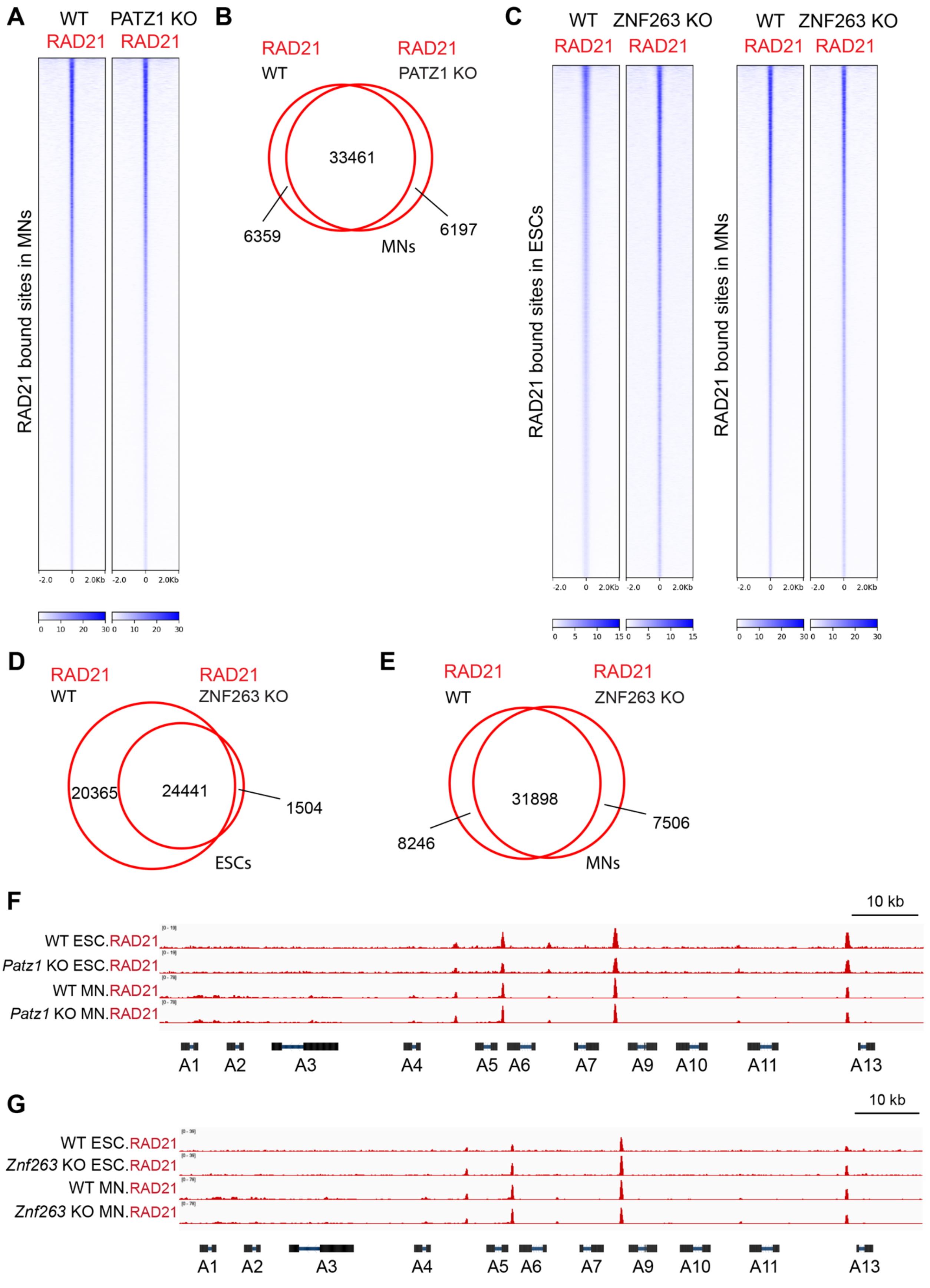
A
WT
PATZ1 KO
RAD21
RAD21
RAD21 bound sites in MNs
B
RAD21
WT
RAD21
PATZ1 KO
33461
6359
6197
MNs
C
WT
ZNF263 KO
RAD21
RAD21
RAD21 bound sites in ESCs
RAD21 bound sites in MNs
D
RAD21
WT
RAD21
ZNF263 KO
20365
24441
1504
ESCs
E
RAD21
WT
RAD21
ZNF263 KO
31898
8246
7506
MNs
F
10 kb
WT ESC.RAD21
Patz1 KO ESC.RAD21
WT MN.RAD21
Patz1 KO MN.RAD21
A1 A2 A3 A4 A5 A6 A7 A9 A10 A11 A13
G
10 kb
WT ESC.RAD21
Znf263 KO ESC.RAD21
WT MN.RAD21
Znf263 KO MN.RAD21
A1 A2 A3 A4 A5 A6 A7 A9 A10 A11 A13

Figure S8. RAD21 binding on chromatin is impacted upon loss of PATZ1 or ZNF263, related to Figure 4 and 5

(A) Heat maps of RAD21 ChIP-seq read density in RAD21 binding sites within a 4 kb window in WT vs PATZ1 KO MNs.

(B) Venn diagram indicating the overlap of RAD21 binding sites in WT vs PATZ1 KO MNs.

(C) Heat maps of RAD21 ChIP-seq read density in RAD21 binding sites within a 4 kb window in WT vs ZNF263 KO mESCs and MNs.

(D) Venn diagram indicating the overlap of RAD21 binding sites in WT vs ZNF263 KO mESCs.

(E) Venn diagram indicating the overlap of RAD21 binding sites in WT vs ZNF263 KO MNs.

(F) Normalized ChIP-seq densities for RAD21 in WT versus *Patz1* KO in mESCs and MNs at the *HoxA* cluster.

(G) Normalized ChIP-seq densities for RAD21 in WT versus *Znf263* KO mESCs and MNs at the *HoxA* cluster.

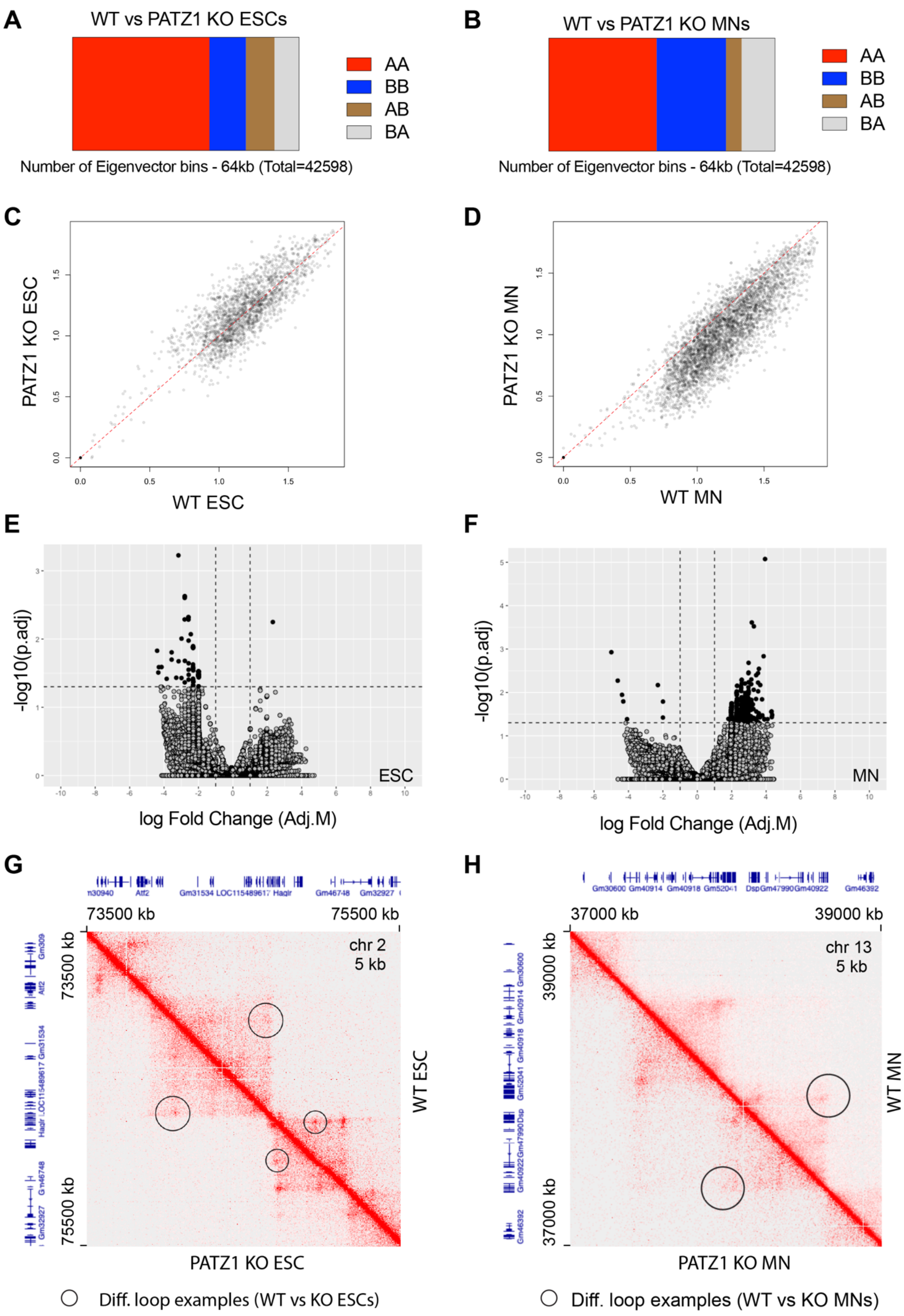
A
WT vs PATZ1 KO ESCs
AA
BB
AB
BA
Number of Eigenvector bins - 64kb (Total=42598)
B
WT vs PATZ1 KO MNs
AA
BB
AB
BA
Number of Eigenvector bins - 64kb (Total=42598)
C
PATZ1 KO ESC
WT ESC
D
PATZ1 KO MN
WT MN
E
-log10(p.adj)
ESC
log Fold Change (Adj.M)
F
-log10(p.adj)
MN
log Fold Change (Adj.M)
G
73500 kb
75500 kb
chr 2
5 kb
WT ESC
PATZ1 KO ESC
Diff. loop examples (WT vs KO ESCs)
H
37000 kb
39000 kb
chr 13
5 kb
WT MN
PATZ1 KO MN
Diff. loop examples (WT vs KO MNs)

Figure S9. Loss of PATZ1 results in changes in the looping interactions at the level of genome organization in ESCs and MNs, related to Figure 6.

(A-B) Bar plot showing AB compartments in WT versus PATZ1 KO ESCs (A) and MNs (B).

(C-D) Scatter plot showing intra-TAD activity scores in WT versus PATZ1 KO ESCs (C) and MNs (D). The scores indicating intra-TAD activity from arrowHead (10 kb) has been plotted in WT versus PATZ1 KO.

(E-F) Volcano plot of differential loop analysis in WT versus PATZ1 KO ESCs (E) and MNs (F). Black circles indicate the values with $p.adj < 0.05$, and gray circles indicate all loops included in the comparative analysis from WT and KO conditions via HiCcompare algorithm (see STAR methods).

(G-H) Visualization of Micro-C contact matrices for a zoomed-in region (G) around the *HoxD* cluster in WT versus PATZ1 KO ESCs, and (H) the *Dsp* locus in WT versus PATZ1 KO MNs. Examples of the differential loops detected are indicated with black circles. The resolution is 5 kb. Shown above and left are gene annotations.

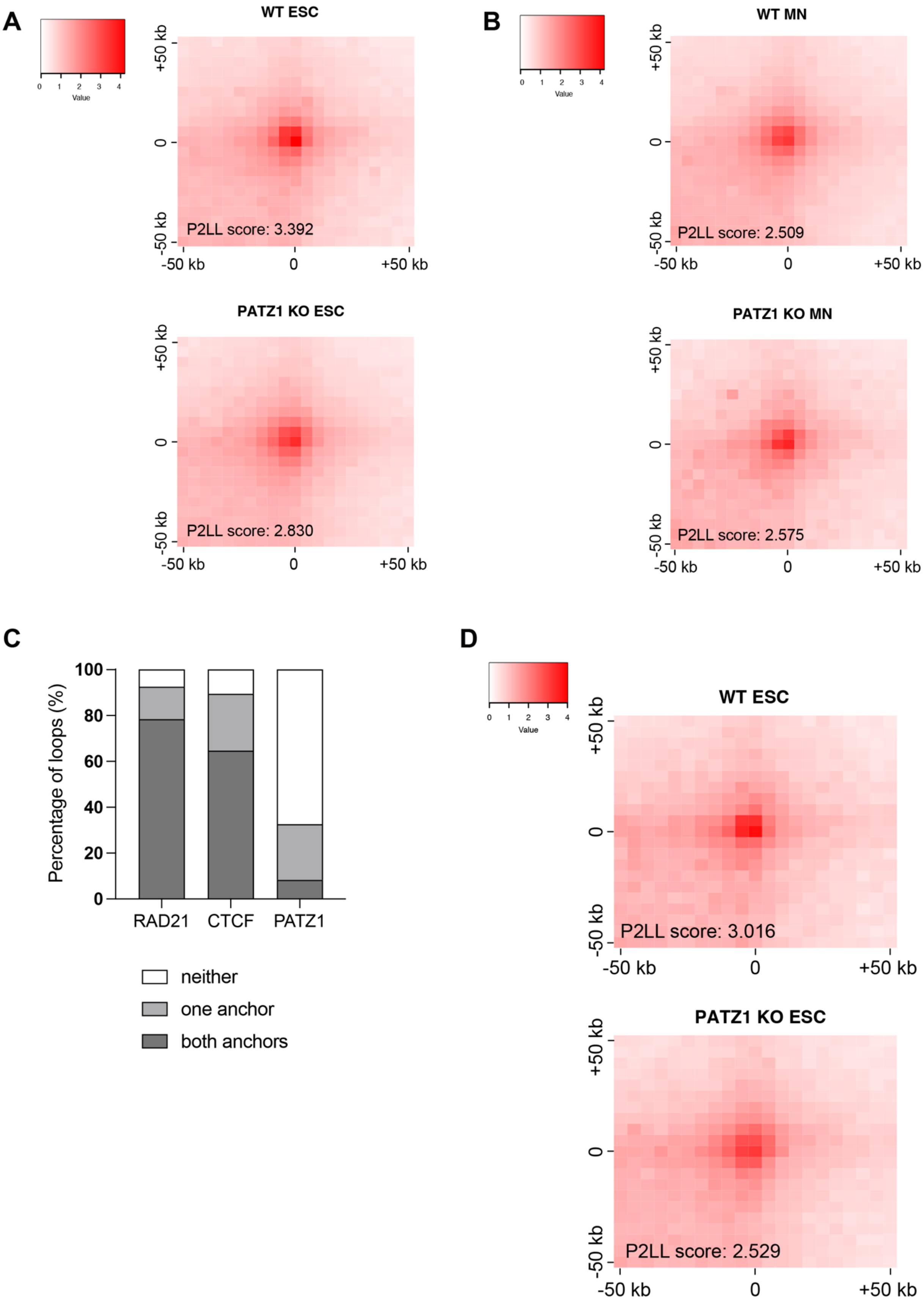
A
WT ESC
P2LL score: 3.392
PATZ1 KO ESC
P2LL score: 2.830
B
WT MN
P2LL score: 2.509
PATZ1 KO MN
P2LL score: 2.575
C
Percentage of loops (%)
RAD21
CTCF
PATZ1
neither
one anchor
both anchors
D
WT ESC
P2LL score: 3.016
PATZ1 KO ESC
P2LL score: 2.529
Value
-50 kb
0
+50 kb

Figure S10. Loss of PATZ1 results in alterations in looping interactions at common loops in ESCs and MNs, and PATZ1-occupied regions in ESCs, related to Figure 6.

(A-B) Normalized APA plots of all common loops between ESCs and MNs plotted in WT versus PATZ1 KO (A) ESCs and (B) MNs. The loops common between WT ESCs and WT MNs have been generated using pgltools as described in STAR methods. The resolution of APA is 5 kb. P2LL (Peak to Lower Left) is the ratio of the central pixel to the mean of the mean of the pixels in the lower left corner (see Table S8 for Micro-C sequencing reads).

(C) Percentage of Micro-C loops in ESCs overlapping with RAD21, CTCF, and PATZ1 ChIP-seq peaks.

(D) Normalized APA plots of loops in WT versus PATZ1 KO ESCs at PATZ1 ChIP-seq co-occupied regions. The resolution of APA is 5 kb. P2LL (Peak to Lower Left) is the ratio of the central pixel to the mean of the mean of the pixels in the lower left corner (see Table S8 for Micro-C sequencing reads).

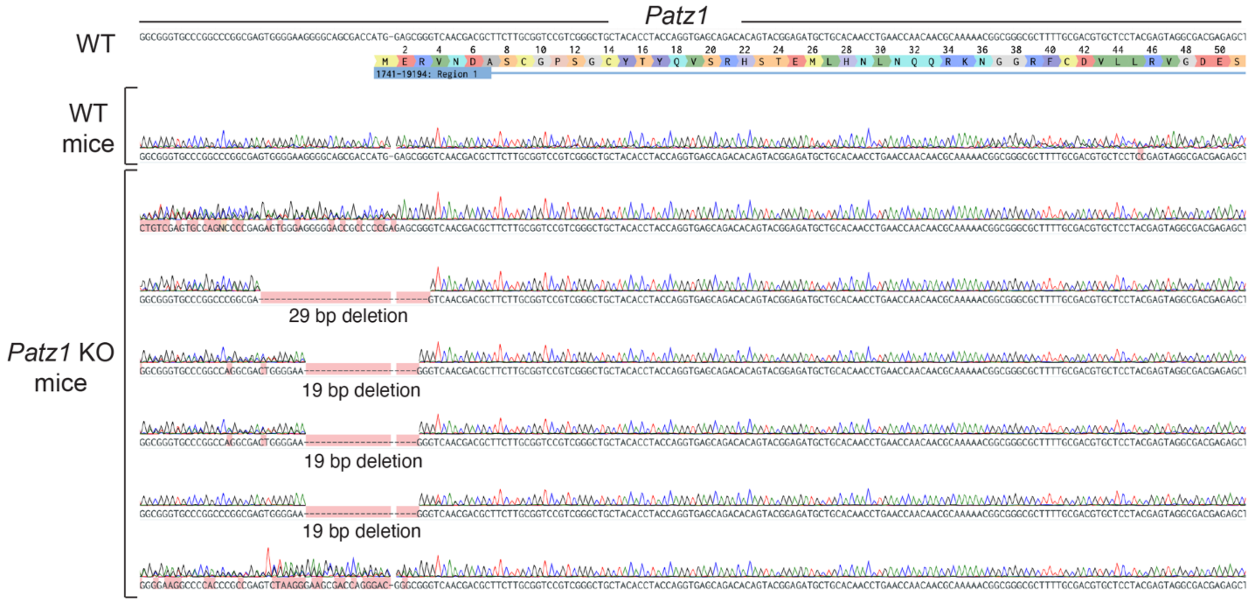

Figure S11. *Patz1* KO mice generation via CRISPR-based zygotic injection *in vivo*, related to Figure 6

CRISPR based deletions in 6 mice upon targeting of the *Patz1* locus (see Figure 6G). 4 mice harbor the indicated deletions resulting in frame-shift mutations, while two mice appear to have heterozygous alleles.

REFERENCES

1. Nora, E.P., Caccianini, L., Fudenberg, G., So, K., Kameswaran, V., Nagle, A., Uebersohn, A., Hajj, B., Saux, A.L., Coulon, A., et al. (2020). Molecular basis of CTCF binding polarity in genome folding. Nat Commun *11*, 5612. 10.1038/s41467-020-19283-x.

2. Ortabozkoyun, H., Huang, P.Y., Cho, H., Narendra, V., LeRoy, G., Gonzalez-Buendia, E., Skok, J.A., Tsirigos, A., Mazzoni, E.O., and Reinberg, D. (2022). CRISPR and biochemical screens identify MAZ as a cofactor in CTCF-mediated insulation at Hox clusters. Nat Genet *54*, 202-212. 10.1038/s41588-021-01008-5.
